# Dietary Inflammatory Index and Disability-Free Survival in Community-Dwelling Older Adults

**DOI:** 10.3390/nu10121896

**Published:** 2018-12-03

**Authors:** Yasutake Tomata, Nitin Shivappa, Shu Zhang, Dieta Nurrika, Fumiya Tanji, Yumi Sugawara, James R. Hébert, Ichiro Tsuji

**Affiliations:** 1 Division of Epidemiology, Tohoku University School of Public Health, Graduate School of Medicine, Sendai 980-8575, Japan; zhangshu@med.tohoku.ac.jp (S.Z.); dieta.nurrika.r4@dc.tohoku.ac.jp (D.N.); f-tanji@med.tohoku.ac.jp (F.T.); yumi1717@med.tohoku.ac.jp (Y.S.); tsuji1@med.tohoku.ac.jp (I.T.); 2 Cancer Prevention and Control Program, University of South Carolina, Columbia, SC 29208, USA. shivappa@email.sc.edu (N.S.); JHEBERT@mailbox.sc.edu (J.R.H.); 3 Department of Epidemiology and Biostatistics, Arnold School of Public Health, University of South Carolina, Columbia, SC 29208, USA; 4 Connecting Health Innovations, LLC, Columbia, SC 29201, USA

**Keywords:** dietary inflammatory index, disability-free survival, disability, cohort

## Abstract

**Background:** Previous studies have reported that a higher dietary inflammatory index (DII^®^) score is related to a higher risk of mortality and conditions that result in functional disability, such as cardiovascular disease, dementia, and fractures. Although these findings suggest that higher DII scores would affect disability-free survival, this has never been investigated directly. The present study investigated the association between the DII score and disability-free survival. **Methods:** We analyzed follow-up data covering a 12-year period for 793 older adults (≥70 years) participating in a Japanese community-based cohort study. DII scores were computed on the basis of dietary intake and assessed using the Brief Self-Administered Diet History Questionnaire. Data on incident functional disability were retrieved from the public Long-Term Care Insurance database. We applied the Cox model for estimating the adjusted hazard ratios (HRs) of the composite outcome (incident functional disability or death) according to DII score tertiles (T1–T3). **Results:** The proportion of men was 47.3%; mean (SD) age was 75.2 (4.5) years. The 12-year incidence of the composite outcome was 65.5%. A higher DII score was related to a higher risk for the composite outcome: HRs (95% confidence interval) were 1.05 (0.84, 1.32) for T2 and 1.26 (1.01, 1.57) for T3 (*p*-trend = 0.040) compared to the most anti-inflammatory T1 reference (HR = 1.00). **Conclusions:** These results suggest that a pro-inflammatory diet might be a modifiable factor affecting disability-free survival in the older population. Additional prospective studies are needed to confirm this relationship.

## 1. Introduction

With the aging of populations worldwide, the corresponding increase in the number of disabled older adults is becoming a significant concern globally [1]. Accordingly, the perceived importance of promoting disability-free life (i.e., extension of independent life span) for older persons has been rapidly gaining attention among researchers in aging-related fields [2].

Also known as “inflamm-aging”, chronic inflammation is associated with aging and chronic conditions such as cardiovascular disease, sarcopenia, dementia, and fracture [3,4]. Because these conditions are known to be causes of incident disability [5], it would be important to control chronic inflammation in order to prevent or minimize any subsequent disability.

Previous studies including meta-analyses of clinical trials have reported that diet is a major modifiable factor associated with inflammation [6,7]. It is known that various nutritional components and food items are associated with inflammation [8]. By unifying the findings of previous studies, the dietary inflammatory index (DII^®^) was developed to estimate the inflammatory potential of the overall diet [8]. The DII has been shown to be associated with inflammatory markers [9,10,11]. In a previous trial, improvement in the DII score after dietary intervention was associated with a reduction of the interleukin-6 level in patients with coronary heart disease [12].

Previous studies have reported that higher DII scores are associated with an increased risk of mortality [13,14] as well as incidence of conditions that can lead to functional disability, such as cardiovascular disease [13,14,15], dementia [16], frailty [17,18], low bone mineral density [19,20], and fractures [20,21]. Although it is expected that higher DII scores (indicating more pro-inflammatory diets) would affect disability-free survival (i.e., not only mortality, but also incident functional disability), this has never been investigated directly. The aim of the present analysis was to investigate the association between pro-inflammatory dietary factors and disability-free survival.

## 2. Materials and Methods

### 2.1. Participants

The Tsurugaya Project was a community-based comprehensive geriatric assessment (CGA) conducted among older Japanese individuals living in Tsurugaya district, Sendai City in northern Japan, in 2003 [22,23]. This comprehensive geriatric assessment (CGA) included a questionnaire-based survey (including dietary habits) as well as clinical examination encompassing physical and cognitive functions.

Among 2925 community-dwelling older individuals aged ≥70 years living in Tsurugaya district, 903 provided informed consent to participate in the Tsurugaya Project, to participate in blood tests for the study, and to review their Long-Term Care Insurance (LTCI) information. Then, we excluded participants who had already been LTCI-certificated as having a disability (*n* = 74) and participants whose energy intakes were considered to lie outside the reasonable range (<2.5 percentile or ≥97.5 percentile) (*n* = 36). Thus, the present study analyzed data from 793 participants.

### 2.2. Dietary Assessment

We assessed dietary habits using a questionnaire, the Brief Self-Administered Diet History Questionnaire (BDHQ) [24]. The BDHQ is a 58-item questionnaire that records the intake frequency of selected foods (but not the portion size) and is designed to estimate the intake volume of food and beverage items during the preceding month. We obtained estimates of the intake volume for food items, energy, and nutrients that were calculated using an *ad hoc* computer algorithm for the BDHQ [24,25]. 

### 2.3. Dietary Inflammatory Index

Based on methods described previously [8,17], the DII score was calculated from 26 food parameters (intake volume) which were available from the BDHQ, i.e., alcohol, vitamin B12, vitamin B6, β-carotene, carbohydrate, cholesterol, energy, total fat, fiber, folic acid, iron, magnesium, monounsaturated fatty acids, niacin, *n*-3 fatty acids, *n*-6 fatty acids, protein, polyunsaturated fatty acids, riboflavin, saturated fat, thiamine, vitamin A, vitamin C, vitamin E, zinc, and tea.

### 2.4. Covariates

To assess the status of social support, participants were asked the following five questions [26]: “Do you have someone (1) with whom you can talk when you are in trouble, (2) whom you can consult when you do not feel well, (3) who can help you with your daily housework, (4) who can take you to a hospital when you feel ill, and (5) who can take care of you if you become bedridden?” For each of these questions, we asked individuals to choose one of two answers: “yes” or “no”. We defined participants who responded “yes” to all five questions as “not lacking” social support (the others were defined as “lacking” social support).

Depressive symptoms were assessed by the Geriatric Depression Scale (GDS). Based on a previous study, we classified individuals with scores of ≥11 as having depressive symptoms [27].

Cognitive function was assessed by the Mini-Mental State Examination (MMSE). Based on a previous study, we classified individuals with scores of ≤26 as having lower cognitive function [28].

The “timed up and go” test measures the time taken to stand up from a chair, walk 3 m, walk back the same way, and sit down again. On the basis of the measured times for this test, we classified the participants (both men and women) into three groups based on sex-specific tertiles.

The number of remaining teeth was assessed by dentists.

The body mass index (kg/m^2^) was calculated from the measured weight and height.

### 2.5. Follow-Up (Incident Functional Disability)

Incident functional disability was defined by the disability certification of the LTCI system. The LTCI is a mandatory social insurance system to assist daily activities of the disabled older adults [29]. Every older person aged ≥65 years is eligible for formal caregiving services. To receive caregiving services through the LTCI system, a person must be certified according to the nationally uniform standard. The procedure for disability certification on the LTCI system comprises two parts: (1) Assessment of the degree of functional disability using a questionnaire designated by the Ministry of Health, Labor, and Welfare, and (2) reference to the Doctor’s Opinion Paper prepared by the attending physician. If a person is judged to be eligible, the Municipal Certification Committee decides on one of seven levels of support, ranging from Support Level 1, Support Level 2, and Care Level 1 to Care Level 5. In general, LTCI certification levels are defined as follows. Support Level 1: “Limited in instrumental activities of daily living but independent in basic activities of daily living (ADLs)”; Care Level 5: “Requiring care in all ADL tasks”. A previous study has shown that the levels of LTCI certification are well correlated with the ability to perform activities of daily living and with the Mini-Mental State Examination score [30]. LTCI certification has already been applied as a measure of incident functional disability in older individuals [22,23].

We also included mortality, certified by the local registry, as an endpoint.

### 2.6. Ethical Issues

We obtained and used information from the baseline survey and follow-up of LTCI certification, after confirming that written consent had been obtained from the participants. The Ethics Committee of Tohoku University Graduate School of Medicine (Sendai, Japan) approved the protocol of this study (approval code: 2002-040).

### 2.7. Statistical Analysis

We counted the person-years of follow-up for each subject from 1 July 2003 until the date of incident functional disability, the date of death, date of moving out, or the end of the study period (30 June 2015), whichever occurred first.

The primary outcome was the composite outcome (incident functional disability or death), as used in previous studies [22,23]. We applied the Cox model to calculate the hazard ratios (HRs) and 95% confidence intervals (CIs) for the composite outcome according to the tertile groups (T1–T3) of the DII score. Dummy variables were created for the tertile groups, and the lowest tertile group was used as the reference group. Multivariate models were adjusted for the following variables. Model 1 was sex- and age-adjusted. Model 2 was further adjusted for history of disease (stroke, hypertension, diabetes, cancer), education level, social support, smoking, depressive symptoms, cognitive function, “timed up and go test” result, and number of remaining teeth. Furthermore, to examine whether macro-nutritional components could explain the association, Model 3 was further adjusted for the body mass index and serum albumin.

Additionally, to examine whether the DII score calculated from our food intake data had validity for the estimation of inflammatory potential, we calculated the Spearman’s correlation coefficient between the DII score and the C-reactive protein level.

All data were analyzed using SAS^®^ version 9.4 (SAS Inc., Cary, NC, USA). All statistical tests described here were two-sided, and differences at *p* < 0.05 were accepted as significant.

## 3. Results

Among the 793 participants, the proportion of men was 47.3%. The mean (SD) age of the entire cohort was 75.2 (4.5) years. The 12-year incidence of the composite outcome was 65.5% (519 cases).

Table 1 compares the characteristics of the participants according to the DII score tertile groups. Participants with a higher DII score were more likely to have an education level of ≤17 years, depressive symptoms, and a longer mean time in the “timed up and go” test. Additionally, participants with a higher DII score were less likely to have ≥20 remaining teeth.

The association between the DII score and the composite outcome is shown in Table 2. A higher DII score was associated with a higher risk of the composite outcome: Adjusted HRs (95% confidence interval) in Model 3 were 1.00 (reference) for T1 (the lowest group), 1.05 (0.84, 1.32) for T2, and 1.26 (1.01, 1.57) for T3 (*p*-trend = 0.040).

The Spearman’s correlation coefficient between the DII score and the C-reactive protein level was ρ = 0.116 (*p* = 0.001) (data not tabulated).

## 4. Discussion

In this cohort study, we investigated the association between the DII score and the composite outcome (incident functional disability or death). We found that the DII score was significantly associated with a higher risk of the composite outcome even after adjustment for potential confounding factors. To our knowledge, this is the first study to have demonstrated an association between the DII score and disability-free survival.

The results did not change substantially even when objective indicators of nutritional status (body mass index and serum albumin) were included in the multivariate model. Therefore, the inverse association seems difficult to explain in terms of only macro-nutritional components (energy and protein), suggesting that other dietary factors (e.g., micro-nutritional components) contribute to the association between the DII score and the composite outcome.

This study has several limitations. First, the sample size was not sufficient to allow for sensitivity analysis. Second, because it was based on the food frequency questionnaire method, which is a memory-based dietary assessment method, we could not rule out the possibility that some of the intake volumes for food items calculated by the BDHQ may have been misclassified. In addition, information on food consumption was obtained only at the baseline. Accordingly, it was unclear whether our DII scores were sufficiently representative of long-term dietary exposure. However, we found a significant positive correlation between the DII score and the C-reactive protein level, suggesting that our DII scores, which are dependent on the food frequency questionnaire data, were indeed valid for the estimation of the inflammatory potential of the diet. Additionally, the method of DII calculation used in the present study was basically consistent with that of a previous study (i.e., 24 food parameters based on the food frequency questionnaire method were applied for DII calculation [17]). Although the strength of the correlation between DII and C-reactive protein was not high (Spearman’s ρ = 0.116), the observed correlation would still be physiologically relevant because even a small improvement in the C-reactive protein level has been shown to be protective against chronic diseases [31]. Indeed, a similar correlation between DII and C-reactive protein was observed in the previous study (Spearman’s ρ = 0.12, *p* < 0.0001), and this DII value was significantly associated with a higher risk of all-cause mortality [32]. Nevertheless, because the present study did not cover some of the potential parameters addressed in the previous study (e.g., flavonoids) [8], there is room for improvement in defining the DII. If it is assumed that a preventive effect would be enhanced by further suppression of chronic inflammation, it would be necessary to define a DII that has a stronger correlation with inflammatory markers. Accordingly, to clarify whether a dietary pattern focused on anti-inflammatory action would be effective for promotion of disability-free survival, a further prospective study using a more valid DII would be necessary.

## 5. Conclusions

This study has shown that a higher DII score was significantly associated with a higher risk of the composite outcome (incident functional disability or death) in older Japanese individuals. These results suggest that a pro-inflammatory diet might be a modifiable factor affecting disability-free survival in the older population. Additional prospective studies are needed to confirm whether the pro-inflammatory diet is a modifiable factor to promote disability-free survival in the older population.

## Figures and Tables

**Table 1:** Baseline characteristics according to dietary inflammatory index groups (*n* = 793).

Characteristics	Dietary Inflammatory Index Tertile			*p* value ^1^
	T1 (Low)	T2	T3 (High)	
*n*	264	264	265	
**Age (years)** ^2^	74.9 ± 4.2	75.5 ± 4.7	75.1 ± 4.4	0.223
**Male Sex (%)**	53.0	43.9	44.9	0.071
**History of (%)**				
Stroke	2.3	3.8	3.4	0.586
Hypertension	40.9	42.8	40.0	0.800
Diabetes	14.8	14.0	16.2	0.770
Cancer	11.4	6.4	9.4	0.140
**Education Level ≤17 years (%)**	26.4	37.6	37.6	0.008
**Social Support Not Lacking (%)** ^3^	67.6	66.5	60.9	0.241
**Current Smoker (%)**	8.5	10.0	13.0	0.226
**Depressive Symptoms (%)** ^4^	22.4	21.0	31.2	0.014
**Lower Cognitive Function (%)** ^5^	12.9	16.7	15.7	0.455
**Timed Up and Go Test Time (s)**	9.0 ± 1.7	9.3 ± 2.1	9.5 ± 2.3	0.010
**Number of Remaining Teeth ≥20 (%)**	52.3	42.4	40.4	0.014
**Body Mass Index (%)**				
<18.5 kg/m^2^	4.9	5.3	6.0	0.294
≥18.5 & <25.0 kg/m^2^	59.5	50.8	57.0	
≥25.0 kg/m^2^	35.6	43.9	37.0	
**Serum Albumin Level (g/dL)**	4.2 ± 0.3	4.2 ± 0.3	4.1 ± 0.3	0.090

^1^ Obtained by using χ^2^ test for variables of proportion and one-way ANOVA for continuous variables. ^2^ Mean ± SD (all such values). ^3^ Participants who considered that they fulfilled all five social support categories. ^4^ Geriatric Depression Scale ≥11. ^5^ Mini-Mental State Examination score ≤ 26.

**Table 2:** Dietary inflammatory index and the composite outcome (*n* = 793).

	Dietary Inflammatory Index Tertile			*p*-trend ^5^
	T1 (Low)	T2	T3 (High)	
**Person-years**	2223	2111	2000	
**Number of Events**	159	174	186	
**Model 1** ^1^	1.00 (reference) ^4^	1.10 (0.89, 1.37)	1.36 (1.10, 1.68)	0.005
**Model 2** ^2^	1.00 (reference)	1.05 (0.84, 1.32)	1.27 (1.02, 1.59)	0.031
**Model 3** ^3^	1.00 (reference)	1.05 (0.84, 1.32)	1.26 (1.01, 1.57)	0.040

^1^ Adjusted for age (continuous value) and sex. ^2^ Adjusted for Model 1 + history of disease (stroke, hypertension, diabetes, cancer), education level (age at final graduation from school ≤17, 18–21, ≥2 years, missing), social support (lack, no lack, missing), smoking (current, former, never, missing), depressive symptoms (Geriatric Depression Scale; ≤10, ≥11, missing), cognitive function (Mini-Mental State Examination; ≤26, ≥27, missing), “timed up and go” test time (sex-specific tertile categories and missing), and number of remaining teeth (≥20, 10–19, <10, missing). ^3^ Adjusted for Model 2 + body mass index (in kg/m^2^: ≤18.4, 18.5–24.9, ≥25.0) and serum albumin (continuous value). ^4^ Hazard ratio (95% confidence interval) of the composite outcome (incident functional disability or death). ^5^ Probability value for trend was computed by entering the categories as a continuous term (DII (dietary inflammatory index) score variable: 1, 2, 3) in the Cox model.
